# Investigation on Synthesis of Hydrogel Starting from Vietnamese Pineapple Leaf Waste-Derived Carboxymethylcellulose

**DOI:** 10.1155/2021/6639964

**Published:** 2021-03-04

**Authors:** Mai Thi Tuyet Phan, Lan Ngoc Pham, Linh Hai Nguyen, Linh Phuong To

**Affiliations:** ^1^Faculty of Chemistry–VNU University of Science, Vietnam National University (VNU), Hanoi, Vietnam; ^2^The Institute of New Technology, Academy of Military Science and Technology, Hanoi, Vietnam

## Abstract

Carboxymethyl cellulose (CMC) is obtained from Vietnamese pineapple leaf waste through etherification. By treating pineapple leaf powder with a solution of NaOH then with HNO_3_ at 90°C for an appropriate time, cellulose can be efficiently extracted. To obtain CMC, carboxymethylation was performed by reaction of the pineapple cellulose with chloroacetic acid at 60°C for 1.5 h. The optimal conditions for this reaction were established. The resulting CMC had a degree of substitution (DS) of 0.91. The hydrogel was prepared by graft copolymerization of acrylic acid and acrylamide to the synthesized CMC. During that reaction, N,N′methylenebisacrylamide (MBA) served as the crosslinking agent and ammonium persulfate (APS) as the initiator. The maximum hydrogel absorbencies for distilled water and 0.9 wt.% NaCl solution were relatively high, namely, 588.2 g/g and 79.3 g/g, respectively. Additionally, the water swelling and water retention behaviors of the hydrogel in soil were also investigated. The results showed that this hydrogel can be employed as a suitable moisture-holding additive in soil for cultivation purposes.

## 1. Introduction

Due to the current water resource crisis, water conservation is essential for the sustainable development of agricultural production. A superabsorbent polymer (SAP) could be an effective solution for water conservation. After absorbing water, SAP particles (also known as hydrogels) act as reservoirs near root systems to store large and abundant amounts of water over periods long enough for plants to grow [1–4]. In the general manufacture of hydrogels, acrylic acid (AA) and acrylamide (AM) are commonly used [5]. However, some weaknesses of these synthetic polymers include their high cost, high toxicity, and poor degradability [6]. The use of polymers of natural origin, such as cellulose, starch, chitosan, xanthan-gum, kappa carrageenan, and locust bean gum [7–11], have recently been considered for copolymerization with hydrophilic synthetic polymers. This shows that this approach is effective and can overcome the abovementioned limitations. In particular, cellulose and its derivatives such as CMC, also referred to as Na-CMC, are attracting much attention from researchers as they are the most abundant source of natural polymers, along with their biocompatibility and biodegradability. Many efforts have been made to synthesize cellulose-based superabsorbent and to improve their swelling capacity. In fact, CMC used as a material to synthesize hydrogel requires a reasonably high solubility and degree of substitution (DS). For CMC to dissolve well, the DS must be high. But a high DS also limits the ability of polymer grafting on CMC. Therefore, research to synthesize CMC with suitable solubility and DS is necessary. Several authors have reported on the synthesis of hydrogels based on CMC from various cellulose sources such as crude cellulose [7], corn husk [12], wood pulp [13], cotton linters [14], etc. It is also known that commercially available CMC is synthesized from cellulose of pure hardwood and cotton. Such manufacturing processes are often complex and the product is expensive. Vietnam is an agricultural country with a large amount of pineapple leaf waste, about 1 M tons annually. Pineapple leaves account for 70% of the total weight of the pineapple and are discarded after the pineapples are harvested. Their release into the environment, in turn, leads to pollution of the living environmental system. As reported in our previous research, cellulose can be extracted from Vietnamese pineapple leaf waste with the relatively high yield of 55% (wt./wt.) [15]. Experiments also showed that the process of separating cellulose from pineapple leaves is quite simple to perform. Cellulose extracted from pineapple leaves appears to have relatively low molecular weight and crystal content as compared to those from wood [7, 13], cotton [14], straw [16], and others [17, 18]. These two factors positively affect the quality of the gel containing cellulose moiety. Thus, with the above advantages, Vietnamese pineapple leaf is an incredibly attractive raw material that needs to be applied to the manufacture of biodegradable superabsorbents (BioSAPs).

So far, most cellulose-based superabsorbent materials have been studied for their absorbance, release capacity of water, and physiological saline solution under laboratory conditions. However, the presence of CMC typically reduced the absorbance of the SAP (without CMC). In many cases, BioSAP's absorbance and release levels in soil conditions have not been evaluated. In fact, cellulose-based superabsorbent materials are easily broken due to weak gel stability under the pressure of the soil resulting in a significant reduction of the water holding capacity of the material [19]. The material's properties sharply decrease due to soil pressure, salt minerals, and fertilizer in the soil. Uncontrolled release of water is one of the main factors limiting the application of this material in agriculture.

The target of this work is to establish the conditions for the synthesis of superabsorbent materials based on pineapple leaf CMC with high water absorption while ensuring consistent gel stability. The obtained products are characterized by their properties and their ability to retain water in soil. In terms of water and physiological NaCl solution absorption, as well as the ability to hold water in the soil, this study highlights the superiority of pineapple leaf-CMC BioSAP compared with the BioSAP containing commercial CMC and SAP (without CMC). This research focuses on two points: (i) the synthesis of CMC from low-cost pineapple leaf waste and (ii) the production of hydrogels based on carboxymethyl cellulose for agricultural application.

## 2. Experimental

### 2.1. Materials and Rice Straw Source

The pineapple leaf waste was collected from the Pineapple Dong Giao Farm, Ninh Binh Province, Vietnam. The leaf samples were dried in an oven at 60°C for 24 h before being ground to powder of 2 mm diameter by using a grinding machine.

The main chemicals used in this study include monochloroacetic acid (MCA) (UK) 99.7%, acetic acid 99.9%, nitric acid 65%, and sodium hydroxyl 99.9% (Merck).

Ammonium persulfate (APS) 99.9%, NaCl 99.9%, and acrylamide (AM) 99.9% were obtained from Merck, N,N-methylenebisacrylamide (MBA) 99.9% was obtained from BioBasic, acrylic acid (AA) 99.6% was obtained from Wako, and NaOH 99% of analytical grade was purchased from Xilong Chemical.

The solvents include methanol 99.8% and ethanol 99.9% from Xilong Chemical, isopropanol 99.7% (Merck), and acetone 99.8% (Merck).

### 2.2. Preparation Methods

#### 2.2.1. Cellulose Recovery from Vietnamese Pineapple Leaf Waste

Cellulose was recovered from Vietnamese pineapple leaves by the chemical method.

In a 1000-ml Becher cup, 10 g of dried pineapple leaf powder was pretreated with 250 ml of diluted 0.75 M NaOH at 90°C for 2 h under stirring. The dark slurry obtained was washed carefully with 250 mL of distilled water to the obtained solid part. The residual solid part was then treated with 150 ml of *y* M HNO_3_ (*y* = 0.250 M, 0.50 M, 0.75 M, 1.00 M, and 1.25 M) and cooked at 90°C for 1.5 h. Next, the mixture was filtered through a no.3 porous funnel and washed several times with distilled water until the pH reached about 7. The obtained residue was dried in an oven at 60°C until the weight no longer changed. The crushed dry product was placed in a closed vial and kept in a desiccator at standard conditions for use in the following test.

The yield of the cellulose extraction was determined by using the following equation:

(1) $$\begin{array}{c} H\left(\%\right)=\frac{m}{m_{0}}\times 100, \end{array}$$

where *m* is the weight of the obtained cellulose and *m*_0_ is the weight of the initial dried pineapple leaf powder.

#### 2.2.2. Synthesis of CMC

The CMC synthesis procedure was conducted according to our previous report [20] with a slight modification. In a 1000 mL Becher cup containing 150 mL of isopropanol, 5 grams of cellulose extraction obtained from Vietnamese pineapple leaf powder was added under stirring for 30 min. Then, 15 mL of (8%, 12%, 16%, and 20%, w/v) NaOH solution was added to the Becher cup and stirred additionally for 1.5 h at 60°C. To start the reaction, *y* g of MCA (*y* = 1.0 g, 2.0 g, 3.0 g, and 4.0 g) was added to the mixture in the cup under additional stirring for 90 min at 60°C. Acetic acid was then used to neutralize the solid part to pH = 7. To remove the byproducts, the product was soaked in 20 ml of ethanol for 10 min and washed. This process was repeated three times. The filtered CMC was dried at 60°C for 2 h and the final product was stored under standard conditions.

The yield of the CMC was determined by equation (1), where *m* is the weight of the obtained CMC and *m*_0_ is the weight of the cellulose used to synthesize CMC.

#### 2.2.3. Preparation of Hydrogel

An aqueous solution of CMC was placed in a 250 mL, four-necked flask equipped with a mechanical stirrer, a thermometer, a reflux condenser, and a nitrogen line to remove dissolved oxygen from the solution. In a separate 200 mL Becher cup, AA was first neutralized to 65% by dropwise addition of a 5 mol/L aqueous NaOH solution to form a mixture of AA and sodium acrylate. The whole process was performed in an ice water bath to avoid the possible polymerization of AA due to the increasing temperature caused by the neutralization reaction. Next, AM and MBA were added to the partially neutralized AA, and they were stirred for 5 min. The obtained solution was then poured into the CMC solution in the flask. The mixture was heated in the oil bath to the desired temperature to start polymerization with constant stirring in a continuous N_2_ line. Finally, the APS solution was added just after the mixture's temperature reached 40°C. The reaction occurred at 60°C for 2 h. The obtained product was cut into small pieces and washed in ethanol twice, soaked in ethanol overnight, and dried in an oven at 60°C for 8 h. The dry product was milled and sieved through a 40-mesh sieve. This product is denoted as BioSAP and the control sample without CMC is denoted as SAP.

### 2.3. Research Methods

#### 2.3.1. Infrared Spectroscopy (FTIR)

Fourier transform infrared spectra were obtained on an FT/IR-6300 spectrometer. The spectral resolution was 4 cm^−1^ and the absorption region was 600–4000 cm^−1^.

#### 2.3.2. X-Ray Diffraction (XRD)

An XRD-6100 model (SHIMADZU) X-ray diffractometer was used to record the X-ray diffraction (XRD) curves of the cellulose and CMC. The diffraction angle ranged from 5° to 80° (0.05^◦^/min). It was recorded with a Cu K target at 30 kV and 15 mA.

#### 2.3.3. Scanning Electron Microscopy (SEM)

The SEM images of the samples were taken on a JSM-5000 model scanning electron microscope at 5.0 kV (Hitachi).

#### 2.3.4. Determination of Degree of Substitution (DS)

DS of CMC was determined according to the Vietnamese standards TCVN 11921-8:2017 [21].

*(1) Sample Preparation.* Five grams of the sample was dissolved in 350 ml of methanol or ethanol in a 500 ml conical flask. The solution was shaken for 30 min then filtered through a porous funnel using a light vacuum. The sample solution was transferred to a crucible and the solvent was evaporated by heating at 100°C for 60 min. Next, the crucible was dried in the oven at 110°C until the weight was unchanged. After each weighing, the crucible was cooled in a desiccator.

*(2) Procedure*. Two grams of the dry substance prepared with the alcohol-extraction procedure mentioned above was weighed in a tared porcelain crucible. First, by a small flame, the crucible was charred carefully. Then, with a larger flame, it was charred for 10 min. Next, the residue was treated with 3–5 ml of concentrated sulfuric acid then heated carefully until no smoke was left. One gram of ammonium carbonate was added while mixing the powder in the crucible thoroughly. Heating was continued with low flame until the smoke stopped. Finally, the crucible was cooled in a desiccator and weighed. The sodium content of the sample was calculated by the following formula:

(2) $$\begin{array}{c} A\left(\%\right)=\frac{a\times 32.28}{b}, \end{array}$$

where *a* is the weight of residual sodium sulfate and *b* is the weight of the alcohol-extracted dry sample.

The degree of substitution was calculated by the following formula:

(3) $$\begin{array}{c} \text{DS}=\frac{162\times A}{2300-80\times A}. \end{array}$$

#### 2.3.5. Viscosity Measurement Method

In this study, viscosity measurements were used to determine the molecular weight of the polymers. For this purpose, the Ubbelohde Capillary Viscometer was used. The intrinsic viscosity was calculated according to the Mark–Houwink–Sakurada equation, namely,

(4) $$\begin{array}{c} \left[\eta \right]=K.M^{\alpha }, \end{array}$$

where [*η*] (dl.g^−1^) is the intrinsic viscosity and *K* and *α* are constants for specific solvents and polymers, respectively. *K* and *α* are 7.3 × 10^−3^ and 0.93, respectively, in 6 wt.% NaOH solution [20, 22].

#### 2.3.6. Determination of Gel Fraction Contents

The gel fraction contents *G* (%) were determined by the Soxhlet technique using acetone as a solvent for 8 h according to equation (1), where *m*_0_ and *m* are the mass of samples before and after Soxhlet technique, respectively.

#### 2.3.7. Determination of Liquid Absorption

The absorption capacity of the synthesized SAPs and BioSAPs was measured in distilled water, tap water, and 0.9 wt. % saline (NaCl) using the tea bag method. This experiment was performed in a standard laboratory. An acrylic-polyester small bag with fine mesh containing 0.2 g of SAP was immersed in 500 mL of water/solution (*t* = 25 ± 2°C, relative humidity, RH = 55 ± 3%). The initial mass of the samples was determined on an analytical balance with 10^−4^ accuracy (*m*_0_). After each designated period, the bag was removed from the solution, drained for 10 min, and weighed (*m*_*t*_). This process was repeated several times until the swelling equilibrium was reached (approximately 24 h), i.e., until the bag presented a constant weight.

The liquid absorption of the samples at each time is determined by the following formula [23, 24]:

(5) $$\begin{array}{c} S\left(\frac{g}{g}\right)=\frac{m_{t}-m_{0}}{m_{0}}, \end{array}$$

where *m*_*t*_ and *m*_0_ are the mass of samples absorbed at time *t* and the mass of the original dried samples, respectively.

The final absorption of the samples was an average of the results.

#### 2.3.8. Determination of Water Retention under Laboratory Conditions

Samples after water absorption saturation were monitored for their release process at 50°C in an oven. After each designated period, the samples were weighed. The water retention of the samples, *R* (%), was determined by equation (1), where *m* and *m*_0_ are the water mass of samples at time *t* and initial time, respectively.

The experiment was conducted at 50^o^C and was repeated 3 times.

#### 2.3.9. Determination of Water Retention in Soil

Two hundred grams of dried soil mixed with 1 g BioSAP was placed into the bottom of a perforated plastic container. The samples no longer received water as long as the first drop of water appeared from the bottom of the box. After each designated interval of time, the samples were weighed. Two hundred grams of control soil sample with no BioSAP was also tested. The water retention in the soil was calculated using equation (7).

The experiment was conducted 3 times at 25 ± 2°C.

## 3. Results and Discussion

### 3.1. Synthesis of CMC from Pineapple Leaf Waste

#### 3.1.1. Cellulose Extraction

The process of cellulose recovery was conducted at various concentrations of HNO_3_ to determine the optimum treatment conditions. The results are listed in Table 1.

In this experiment, HNO_3_ was used to extract cellulose from the solid residual part in the previous alkaline pretreatment stage and the yield of cellulose reached maximum at 0.75 M HNO_3_. It also can be seen in Table 1 that with higher levels of HNO_3_ concentration, namely, of 1.00 M and 1.25 M, the yield of cellulose decreases gradually. This might be due to the destruction of the cellulose structure at high concentrations of HNO_3_. In brief, the highest yield of the cellulose extraction was 51.13 ± 4.17% at 0.75 M HNO_3_.

#### 3.1.2. CMC Synthesis

*(1) Effect of NaOH Concentration on DS and Yield of CMC.* The goal of using NaOH as a reagent is to swell cellulose chains and provide the possibility to substitute carboxyl groups by sodium carboxymethyl groups in the cellulose units. The DS of the CMC obtained at different concentrations of sodium hydroxide is shown in Table 2.

As shown in Table 2, the DS of the CMC increased with NaOH concentration and attained the highest DS of 0.91 at a NaOH concentration of 16% (w/v). However, further increase of NaOH concentration leads to a reduction in DS values. When sodium hydroxide enters the cellulose molecules to react with the -OH groups, the morphology of the cellulose changes. Specifically, crystalline regions transform into amorphous ones and allow carboxymethylation to occur [22]. However, the morphology conversion only occurs to a finite level as it also depends on other factors such as solvents and reagents. On the other hand, during the carboxymethylation, a reaction between MCA and NaOH could occur. When the NaOH concentration is too high, this reaction trend dominates and leads to a decrease in the possibility of an etherification reaction. These results are similar to those of Chumee [25] and Sunardi [26]. Table 2 also shows the CMC yields at different NaOH concentrations, which had similar trends as the DS results.

*(2) Effect of MCA Weight on DS and Yield of CMC.* The effect of MCA weight on the DS value was determined by changing the amount of MCA from 1.0 g to 4.0 g. The result is shown in Table 3, where the DS of the CMC increased with an increasing amount of MCA in a range of 1.0–4.0 g and then decreased slightly with further increase of MCA. The highest DS value was observed at an MCA weight of 3.0 g. This may be due to the fact that an undesired side reaction occurred at high MCA amounts, which leads to a reduction in CMC yield. This similar range of DS values (0.58–0.91) was also shown in another report [25] for pomelo peel waste. Table 3 also shows that the trend in CMC yield is similar to that of the DS.

Thus, the optimum condition for carboxymethylation was 5 g cellulose, 3.0 g chloroacetic acid, and 15 ml of 16%w/v NaOH solution. The obtained CMC had a DS of 0.91.

### 3.2. Structural Characterization

**Figure 1:**
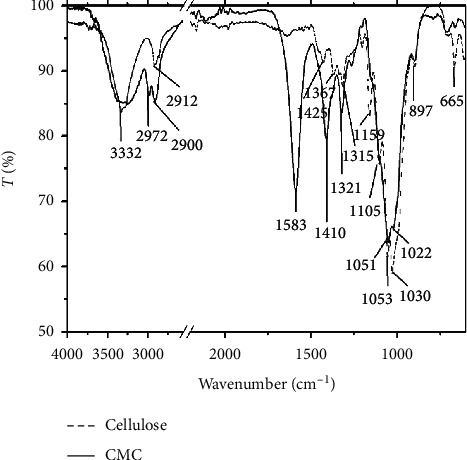
FTIR spectroscopy of extracted cellulose and synthesized CMC.

FTIR spectroscopy was used to confirm the structure of the extracted cellulose and synthesized CMC (see Figure 1). It can be seen from the FTIR spectra of cellulose that the absorption peak at 3332 cm^−1^ can be assigned to the OH-stretching vibrations, while the signals at 2912 cm^−1^ and 1315 cm^−1^ are characteristic of deformation vibrations of the C-H groups in the glucose units. The 1159 cm^−1^ peak corresponds to the stretching vibrations of the -C-O-C group in the *β*-(1,4)-glycosidic linkages of the cellulose molecules. The peak at 1105 cm^−1^ corresponds to the -C-O group of the secondary alcohols and ethers in the cellulose backbones. Finally, the absorption band at 897 cm^−1^ corresponds to the *β*-(4,1)-glycosidic linkages between the glucose units [25].

The FTIR spectrum of the cellulose extracted from Vietnamese pineapple leaf waste looks similar to that in a study of López et al. [27]. In addition, the absence of peaks at 1600–1800 cm^−1^, characteristic of the functional groups C=O and the aromatic ring of hemicellulose and lignin molecules [26, 28], proved that hemicellulose and lignin were completely removed. This means that the recovered cellulose is of high purity. This pure cellulose was then used for CMC synthesis.

Absorption peaks around 3332 cm^−1^, belonging to OH-stretching vibrations, can be seen in Figure 1. These peaks are broad because of the intermolecular and/or intramolecular hydrogen bonds existing in cellulose. The bands at 2972 cm^−1^ and 2900 cm^−1^ are attributed to the stretching vibrations of the C-H groups. The peaks at 1051 cm^−1^ and 1022 cm^−1^ are relevant to the *β*-(1,4)-glycosidic linkages between the glucose units in cellulose [28, 29]. In the CMC spectrum, the strong absorption at 1583 cm^−1^ and 1410 cm^−1^ was attributed to C=O stretching and confirmed the presence of the -COO and -COONa groups, which indicate the successful etherification of cellulose. These peaks do not exist in the FTIR spectroscopy of cellulose (see Figure 1). The above analysis results are similar to those of earlier publications of Chumee [25] for pomelo peel waste, Sunardi [26] for purun tikus, and S. Sophonputtanaphoca [16] for Thailand pineapple leaves.

**Figure 2:**
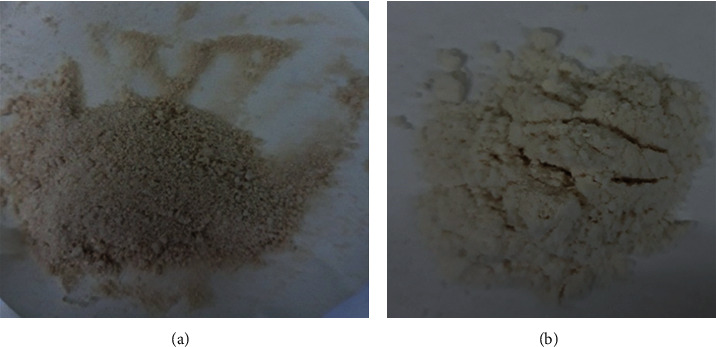
Images of pineapple leaves cellulose (a) and CMC (b).

**Figure 3:**
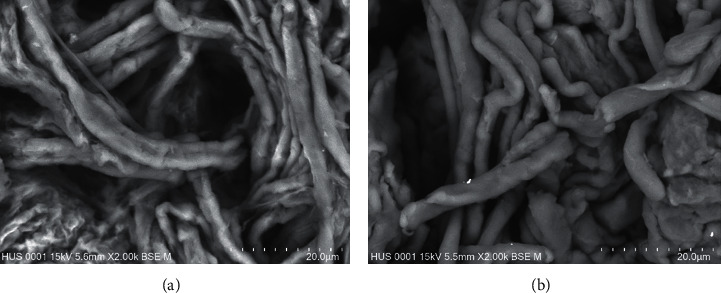
SEM images of pineapple leaves cellulose (a) and CMC (b). Scale bars represent 20 *μ*m (magnification = 2000*x*).

The microstructure and morphology of the materials were observed by using SEM.  Figure 2 shows photographs of the extracted cellulose and that of CMC. The SEM images of both products are shown in Figure 3.

Cellulose and synthesized CMC showed a ribbon shape or rod-like morphology. The same morphology has been observed by other authors in their research [16, 27]. It can also be seen from Figure 3 that the surfaces of the extracted cellulose yarns were smooth and had very low damage. Meanwhile, for the CMC, the surface was more extended, rough, and collapsed. This is due to the fact that the extracted cellulose was treated with sodium hydroxide during carboxymethylation. Figure 3 also shows that the size of cellulose is in the range of 2.0–3.0 *μ*m and that of CMC is 2.5–3.5 *μ*m.

**Figure 4:**
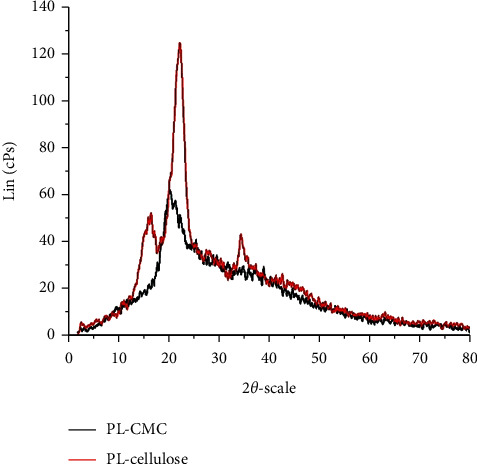
X-ray diffractogram of isolated cellulose (red curve) and CMC (black curve) from pineapple leaf waste.

In this study, the degree of crystallinity before and after the carboxymethylation of cellulose was investigated by XRD [12,16]. The X-ray diffractograms of isolated cellulose and CMC from pineapple leaf waste are shown in Figure 4. It can be inferred from Figure 4 that CMC is less crystalline than holocellulose. More specifically, the XRD curve of holocellulose contains three peaks at 2*θ* = 16.3, 22.7, and 34.6 deg and the two peaks at 2*θ* = 16.3 and 22.7 deg are sharp, meaning that the core structure of holocellulose has more crystalline phases. The diffractogram of CMC showed less peaks in comparison with that of holocellulose and the peak intensity was significantly smaller. The cause of this is the presence of more amorphous structures in CMC compared to holocellulose. From the above observation, we could assume that CMC would have disordered molecular arrangement in comparison with isolated holocellulose. The reason for this is the presence of carboxymethyl moieties in CMC, which is a product of carboxymethylation.

**Figure 5:**
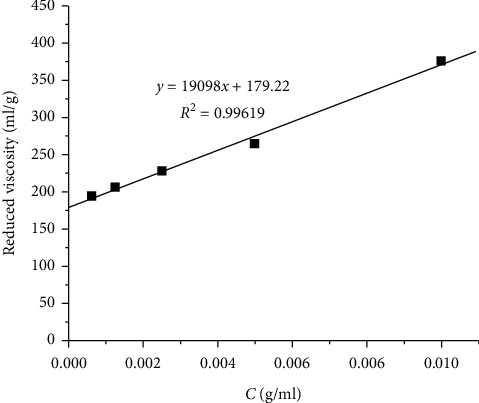
Mark–Houwink–Sakurada plot for CMC in 6% wt. NaOH at 25°C.

The average molecular weight (*M*) is an important parameter of carboxymethyl cellulose. It affects swelling, the solubility of CMC in the water, its structure, and other properties. Intrinsic viscosity [*η*] can be obtained by extrapolation of reduced viscosity [*η*_red_] to zero concentration (see Figure 5) as in the following equation:

(6) $$\begin{array}{c} \left[\eta \right]=\underset{C⟶0}{\lim}\frac{\eta_{\text{sp}}}{C}=\underset{C⟶0}{\lim}\eta_{\text{red}}, \end{array}$$

where *η*_*r*_ is reduced viscosity, *η*_sp_ is specific viscosity, and *η*_sp_ = *η*_*r*_ – 1.

The intrinsic viscosity in relation with average molecular weight is represented by Mark–Houwink–Sakurada empirical equation:

(7) $$\begin{array}{c} \left[\eta \right]=K.M^{\alpha }. \end{array}$$

The Mark–Houwink constant, *K*, and *α* for CMC were 7.3 × 10^−3^ ml/g, and 0.93, respectively.

The [*η*] values can be estimated from the intercept of the plot (Figure 5), which was [*η*] = 179.22 (ml/g). The average molecular weight of CMC is 52.535 ± 251 g/mol.

CMC product was used to synthesis superabsorbent polymer.

### 3.3. Study on Synthesis Conditions of BioSAP

#### 3.3.1. Synthesis of BioSAP

**Figure 6:**
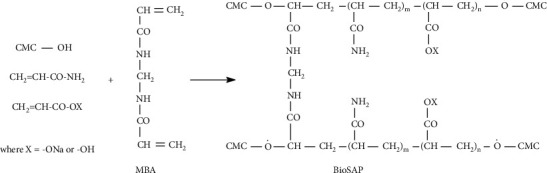
Reaction scheme of BioSAP synthesis.

The BioSAP synthesis reaction is a graft copolymerization reaction. The reaction scheme is presented in Figure 6.

In this reaction, polyacrylate and/or polyacrylamide are grafted onto CMC to form polymer chains. The polymer chains are cross-linked with one another via N,N′-methylenebisacrylamide (MBA). The crosslink density affects the gel strength and gel absorption capacity.

#### 3.3.2. Characterization of BioSAP

The properties of the BioSAP synthesized in the presence of 1% APS, 0.1% MBA, and 10% CMC by weight are studied.

**Figure 7:**
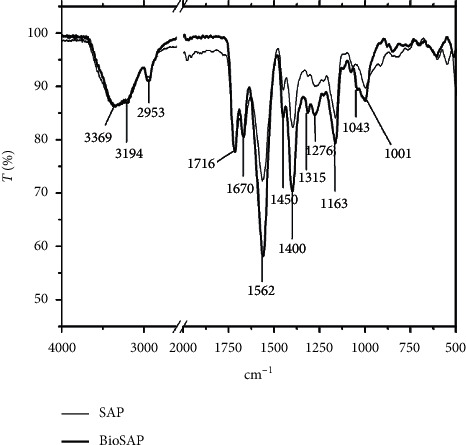
FTIR spectrum of SAP and BioSAP samples.

*(1) FTIR Spectrum.* The FTIR spectra of the SAP and BioSAP samples are shown in Figure 7.

As can be seen from the FTIR spectra of the BioSAP, the peaks appearing at 3369 cm^−1^ and 3194 cm^−1^ are typical of the valence vibrations of the O-H and N-H bonds, respectively, and the peak at 2953 cm^−1^ represents the valence vibrations of the C-H bonds. The appearance of peaks at 1716 cm^−1^ and 1670 cm^−1^ characterizes the vibrations of the C=O bonds of acids and amides, respectively. In particular, the peak at 1562 cm^−1^ is typical for a sodium carboxylate salt [5–7]. The peaks at 1400 cm^−1^, 1315 cm^−1^, and 1276 cm^−1^ characterize the vibrations of the C-N, C-H, and C-C bonds, respectively. The increase in peak intensity of BioSAP compared to SAP at 1562 cm^−1^, 1163 cm^−1^, and 1001 cm^−1^ in the FTIR spectrum is due to the appearance of the C=O bonds of carboxymethyl groups and the C-O-C bonding bridge between the glucoside rings and CH-*β*-glycoside of CMC [16, 17]. Thus, the analysis of FTIR spectrum demonstrates the presence of CMC in the structure of BioSAP.

*(2) Gel Fraction Contents*. The refinement of raw products has been carried out by the Soxhlet technique, which removes impurities such as water-soluble homopolymers, oligomers, residual monomers, catalysts, and others. The gel fraction of the products reached 98.5%. The saturated absorption of distilled water (*S*_DW_), tap water (*S*_TW_), and 0.9% NaCl solution (*S*_NaCl_) of the raw and refined products is given in Table 4.

The results of Table 4 show that the refined BioSAP samples greatly improved the absorption for all the media tested. Thus, the refinement of the products after synthesis is very important. The absorption of tap water and 0.9% NaCl solution is significantly lower than that of distilled water, which proves that the presence of metal ions has a great influence on the water absorption capacity of the BioSAP materials. The high water absorption of the pineapple cellulose-BioSAP (nearly 560 g/g) is very impressive. This value is higher than that of the BioSAP-containing commercial CMC [7, 30] and much higher than that of SAP (with no CMC) [5, 30]. Additionally, as shown in the study of BioSAP's water holding capacity in soil, the water retention and slow-release capacity of our BioSAP is significantly higher than that of other published studies [1–4]. This is even more impressive when taking into account that pineapple cellulose-BioSAP has a definitively lower cost than the others as pineapple leaf is a waste material.

#### 3.3.3. Survey on Content of CMC

**Figure 8:**
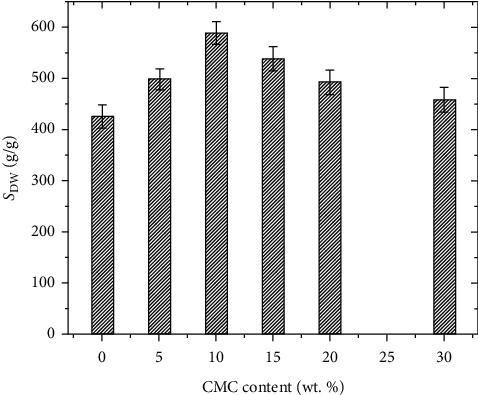
Absorption of distilled water of BioSAP with different CMC content.

The BioSAP samples were prepared with varying amounts of CMC (from 0 to 30%) in the presence of 1% APS and 0.1% MBA by mass. The relationship between CMC content and the water absorption of BioSAP is shown in Figure 8.

From Figure 8, it can be seen that increasing CMC content leads to an increase in the distilled water absorption of BioSAP, which reaches a maximum at 10% CMC. Then, further increase of CMC content causes the water absorption to gradually decrease. This can result from the many hydrophilic groups, such as -OH and -COO^−^, contained in CMC along its chains. In addition, CMC chains act as a framework for grafting polyacrylic acid, polyacrylate, and polyacrylamide chains [5–7]. Thus, the increase of CMC content means creating more grafting sites, which results in more polymers branching in and a higher absorption ability of the material. Thus, upon increasing the content of CMC, more space in the material is created and a greater number of hydrophilic functional groups appear, which lead to an increase in the water absorption. Specifically, the presence of CMC at a loading of 10 wt. % significantly increases the water absorption of copolymer (acrylic acid-co-acrylamide) from 426.1 ± 22.2 up to 588.2 ± 21.8 g/g. This is due to the CMC molecules acting as backbones to make graft copolymers, thereby increasing hydrogel strength and helping them to retain their structure during the absorbing process, which enhances water absorption ability. So, this result indicates that this suitable CMC content provides a compromise between the absorbency and structure stability of the gel.

However, further increase of the amount of CMC reduces both the water absorption capacity and gel stability. This decrease could be due to the fact that high content of CMC acts as a filler that reduces the empty space in the BioSAP for water storage. This interpretation is supported by the result in [5]. Additionally, in higher CMC content media, the BioSAP gel was observed to be weaker (low gel strength) and the materials became sticky. While attempts were made in this study to synthesize SAP with CMC content higher than 40%, the obtained material dissolved in water when swelling experiments were performed for longer than 48 h. This suggests that too high CMC content makes the crosslinking density insufficient and results in a loose gel structure. In other words, the gel strength was too weak to hold water molecules. It is known in the literature that CMC increases biodegradability [3, 4]. So, depending on the material's requirements for water absorption and self-degradation time, CMC content can be selected between 5 and 20%. In this work, the main purpose of the introduction of CMC into the superabsorbent was to increase the water absorption while ensuring consistent gel stability. Thus, 10% CMC is the most suitable content. This BioSAP material was characterized in terms of chemical structure and gel fraction content. Its adsorption-desorption behavior in solutions was also studied. Finally, this BioSAP was tested on the water holding capacity in the soil.

The effect of CMC on liquid adsorption-desorption behavior of SAP materials.

**Figure 9:**
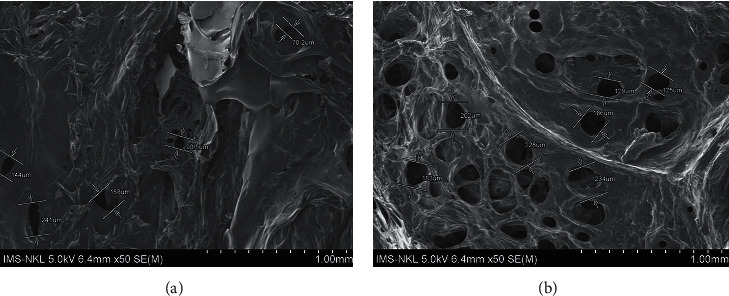
SEM images with 50x magnification of SAP (a) and BioSAP. (b) Scale bars represent 1 mm (magnification = 50x). It can be seen from Figure 9 that, in the presence of CMC, the hydrogel material has many pores.

From Figure 8, we can see that the presence of CMC in the material's structure significantly increases the water absorption from 426.1 ± 22.2 up to 588.2 ± 21.8 g/g. The increase in water absorption of the material can be explained by the fact that CMC molecules contain many hydrophilic groups such as –OH and -COO^−^ along their chain length. At the same time, CMC molecules act as a backbone to make graft copolymers and increase hydrogel strength by helping them retain their structure during the absorbing process, which enhances the water absorption ability. In addition, the participation of CMC molecules in copolymer macromolecules also increases pore size and leads to an enhanced water absorption capacity of the material [8, 16, 17]. These results are also corroborated by SEM image in Figure 9.

Notably, the pore structure of the CMC-containing hydrogel has many subcavities, which may be the cause of its high water absorption, as stated in our goal.

**Figure 10:**
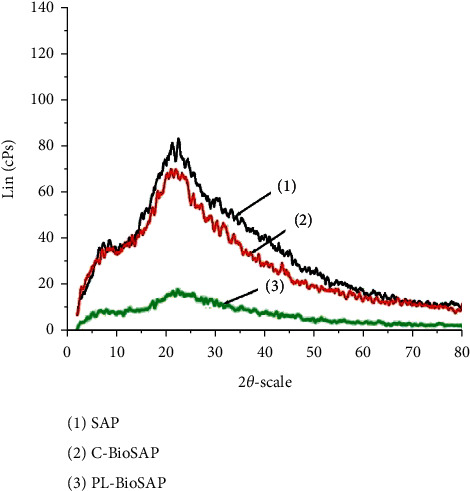
XRD curves of SAP and BioSAP. Curve 1: SAP with no CMC; curve 2: C-BioSAP with commercial CMC; and curve 3: PL-BioSAP with pineapple leaf CMC.

It is worth mentioning that the water absorption value of this BioSAP was significantly higher than that of the synthetic commercial CMC-based BioSAP reported in our previous work [30], i.e., 558.2 ± 21.8 g/g versus 310 ± 18.2 g/g. This result revealed that the state of CMC significantly affects the properties of the hydrogel. This result is also proven by the XRD study of SAP and BioSAP as shown in Figure 10.

It can be seen from the XRD plot of Figure 10 that the hydrogel sample without CMC (SAP, curve 1) has a peak at 2*θ* = 23.5 deg. The presence of commercial CMC (curve 2) contributed to the breakdown of the crystal structure of SAP and increased the amorphous region of the material, as indicated by a decrease in peak intensity at 2*θ* = 23.5 deg. Notably, as expected, the peak intensity of the pineapple leaf CMC-based BioSAP (curve 3) decreased dramatically. That is, pineapple leaf CMC broke down almost completely the crystal structure of the SAP substrate. This helps to explain the remarkable increase in the water absorption of pineapple CMC-based BioSAP compared with BioSAP containing commercial CMC, as well as with SAP.

**Figure 11:**
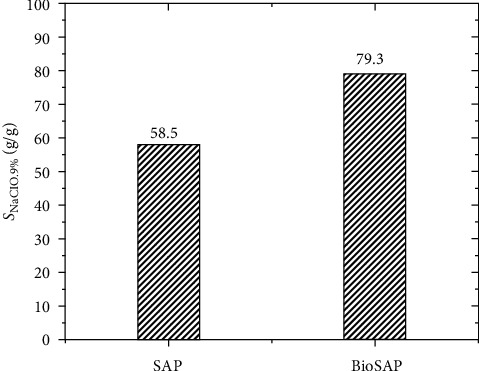
NaCl 0.9% absorption of BioSAP containing 10% CMC and of SAP.

**Figure 12:**
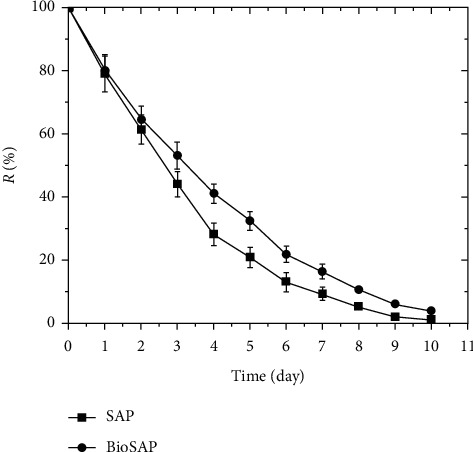
Water retention of BioSAP and SAP at 50°C.

Notably, the presence of CMC also significantly improved the 0.9 wt.% NaCl solution absorption of the BioSAP materials with 79.3 g/g compared to 58.5 g/g of the SAP (see Figure 11). It should also be added that the absorption value of the physiological saline solution (79.3 g/g) of this BioSAP is much greater than that of the SAPs of other studies [5–9]. Good water absorption is an important property for the application of this material to the agricultural sector as it helps to increase water absorption in the soil environment. In this study, the effect of CMC on the desorption behavior of SAP materials is also investigated. The result is shown in Figure 12.

It can be seen that the presence of CMC reduces the rate of water release of the materials, which implies that CMC increases the water holding capacity. For example, after 3 d at 50°C, the polymer sample containing CMC (BioSAP) held up to 53.34% of the water absorbed while the sample without CMC (SAP) held only 44.23%. This may be explained by the presence of a large number of hydrophilic groups such as -OH and -COO^−^ along the molecular backbone of CMC chains, which form hydrogen bonds with water molecules and reduce the probability of water release in the material. Thus, the addition of CMC improved the absorption of water and 0.9% NaCl solution while also increasing the water holding capacity of these BioSAP materials.

#### 3.3.4. Test on the Water Holding Capacity of BioSAP in Soil

**Figure 13:**
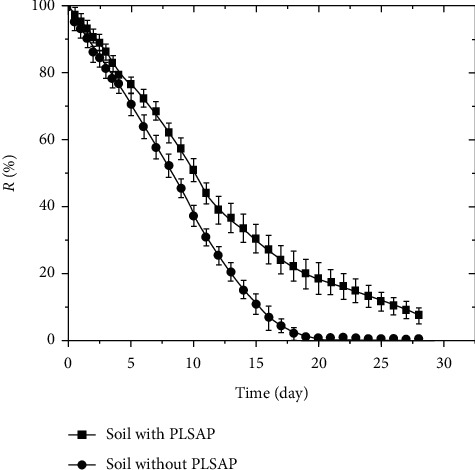
Water retention in soil with and without 0.5% BioSAP by mass.

To assess the water holding capacity of BioSAP in soil, water retention and slow-release tests were conducted, and the results are shown in Table 5 and Figure 13.

It can be seen from Table 5 that the volumes of water retained in the soil samples containing BioSAP are almost 3 times greater than those held in the soil samples without BioSAP.

As can be seen from Figure 13, soil samples containing 0.5% BioSAP have a significantly higher ability of water retention than those without BioSAP. Over the first 10 days, the amount of water absorbed decreased sharply and then decreased gradually. After 15 days, samples with BioSAP could still hold 33% of water while those without BioSAP could hold only 10%. After 28 days, the water in the soil sample without BioSAP completely evaporated, while the soil samples with BioSAP retained nearly 7.5%. This proves that BioSAP improves the water holding time in the soil as well as the water release time from the soil. Notably, water retention and the slow-release capacity of our BioSAP products are significantly higher than those of other published studies [1–4].

## 4. Conclusions

Cellulose was successfully extracted from Vietnamese pineapple leaf waste with a maximum cellulose extraction yield of 51.13 ± 4 wt.% by dried mass. CMC (DS of 0.91 and mol mass of 52.535 ± 251 g/mol) was obtained by etherifying the obtained cellulose with monochloroacetic acid. Hydrogels based on pineapple leaf CMC (BioSAP) were revealed to have superior water and 0.9% NaCl solution absorption abilities compared to the commercial CMC-based hydrogel. It was shown that by using 0.5 wt. % soil volume of BioSAP based on pineapple leaf waste as a soil moisturizer, the water holding capacity increased more than 3.5 times and the water release time lengthened from 15 days to 28 days.

This research also shows, from a technological and economic perspective, that the fabrication of BioSAP from discarded pineapple leaf waste in Vietnam has immense potential and is quite feasible. It is also very beneficial in terms of environmental protection.

## Figures and Tables

**Table 1:** Cellulose yield with various HNO_3_ concentrations.

Yield of cellulose	C_HNO3_, M				
	0.25	0.50	0.75	1.00	1.25
*H*_*c*_ (%)	32.50 ± 2.12	49.50 ± 3.05	51.13 ± 4.17	39.20 ± 5.76	36.21 ± 6.79

**Table 2:** The yield and DS of synthesized CMC with various NaOH concentrations.

	NaOH, %wt.			
	8	12	16	20
*H*_CMC_, %	162.1	170.3	178.8	172.6
DS	0.64	0.72	0.91	0.86

**Table 3:** The yield and DS of CMC synthesized with various amounts of MCA.

	Amount of MCA, g			
	1.0	2.0	3.0	4.0
*H*_CMC_, %	153.7	172.3	178.8	169.1
DS	0.58	0.71	0.91	0.79

**Table 4:** Saturated absorption of BioSAP samples.

Samples	*S*_DW_, g/g	*S*_TW_, g/g	*S*_NaCl_, g/g
Raw BioSAP	525.0	313	71.0
Refined BioSAP	588.2	375	79.3

**Table 5:** Water retention of soil samples with and without BioSAP.

Samples	*m*_BioSAP_,g	*m*_soil_,g	*m*_water_,g
Soil with BioSAP	1.0	200 ± 0.1	131 ± 5.5
Soil without BioSAP	0.0	200 ± 0.1	36 ± 2.6

## Data Availability

The data used to support the findings of this study are available from the corresponding author upon request.

## References

[1] Waleed A. (2018). Impact of hydrogel polymer in agricultural sector. *Advances in Agriculture and Environmental Science*.

[2] Demitri C., Scalera F., Madaghiele M., Sannino A., Maffezzoli A. (2013). Potential of cellulose-based superabsorbent hydrogels as water reservoir in agriculture. *International Journal of Polymer Science*.

[3] Montesano F. F., Parente A., Santamaria P., Sannino A., Serio F. (2015). Biodegradable superabsorbent hydrogel increases water retention properties of growing media and plant growth. *Agriculture and Agricultural Science Procedia*.

[4] Stahl J. D., Cameron M. D., Haselbach J., Aust S. D. (2000). Biodegradation of superabsorbent polymers in soil. *Environmental Science and Pollution Research*.

[5] Suo A., Qian J., Yao Y., Zhang W. (2007). Synthesis and properties of carboxymethyl cellulose-graft-poly(acrylic acid-co-acrylamide) as a novel cellulose-based superabsorbent. *Journal of Applied Polymer Science*.

[6] Pourjavadi A., Ghasemzadeh H., Mojahedi F. (2009). Swelling properties of CMC-g-poly (AAm-co-AMPS) superabsorbent hydrogel. *Journal of Applied Polymer Science*.

[7] Pairote K., Patchareeya K. (2017). SAP based on sodium carboxymethyl cellulose grafted polyacrylic acid by inverse suspension polymerization, synthesis and property of SAPs modified by carboxymethyl cellulose. *International Journal of Polymer Science*.

[8] Alam M. N., Islam M. S., Christopher L. P. (2019). Sustainable production of cellulose-based hydrogels with superb absorbing potential in physiological saline. *ACS Omega*.

[9] Pandey S., Ramontja J. (2016). Natural bentonite clay and its composites for dye removal: current state and future potential. *American of Journal Chemistry and Applications*.

[10] Pandey S. (2017). A comprehensive review on recent developments in bentonite-based materials used as adsorbents for wastewater treatment. *Journal of Molecular Liquids*.

[11] Pandey S., Do J. Y., Kim J., Kang M. (2020). Fast and highly efficient removal of dye from aqueous solution using natural locust bean gum based hydrogels as adsorbent. *International Journal of Biological Macromolecules*.

[12] Mondal M. I. H., Yeasmin M. S., Rahman M. S. (2015). Preparation of food grade carboxymethyl cellulose from corn husk agrowaste. *International Journal of Biological Macromolecules*.

[13] Zampano G., Bertoldo M., Bronco S. (2009). Poly(ethyl acrylate) surface-initiated ATRP grafting from wood pulp cellulose fibers. *Carbohydrate Polymers*.

[14] Haleem N., Arshad M., Shahid M., Tahir M. A. (2014). Synthesis of carboxymethyl cellulose from waste of cotton ginning industry. *Carbohydrate Polymers*.

[15] Phan T. T. M., Pham T. H. (2019). Potential biogas production from wasted pineapple leaves. *Chemistry of Journal*.

[16] Sophonputtanaphoca S., Chutong P., Cha-aim K., Nooeaid P. (2019). Potential of Thai rice straw as a raw material for the synthesis of carboxymethylcellulose. *International Food Research Journal*.

[17] Kabir S. M. F., Sikdar P. P., Haque B., Bhuiyan M. A. R., Ali A., Islam M. N. (2018). Cellulose-based hydrogel materials: chemistry, properties and their prospective applications. *Progress in Biomaterials*.

[18] Dai H., Huang H. (2016). Modified pineapple peel cellulose hydrogels embedded with sepia ink for effective removal of methylene blue. *Carbohydrate Polymers*.

[19] Lejcus K., Spitalniak M., Dąbrowska J. (2018). Swelling behavior of SAPs for soil amendment under different loads. *Polymers*.

[20] Phan T. T. M., Ngo T. S. (2020). Pectin and cellulose extraction from passion fruit peel waste. *Vietnam Journal of Science, Technology and Engineering*.

[21] (2009). COEI-1-CMC: 2009, Carboxymethylcellulose (cellulose gum). *International Œnological Codex*.

[22] Zhang S., Li F., Yu J., Gu L.-X., Zhang S. (2009). Disolved state and viscosity properties of cellulose in a NaOH complex solvent. *Cellulose Chemistry and Technology*.

[23] Sabbagh F., Khatir N. M., Karim A. K., Omidvar A., Nazari Z., Jaberi R. (2019). Mechanical properties and swelling behavior of acrylamide hydrogels using montmorillonite and kaolinite as clays. *Journal of Environmental Treatment Techniques*.

[24] Sabbagh F., Muhamad I. I. (2017). Acrylamide-based hydrogel drug delivery systems: release of acyclovir from MgO nanocomposite hydrogel. *Journal of the Taiwan Institute of Chemical Engineers*.

[25] Chumee J., Seeburin D. (2014). Cellulose extraction from Pomelo peel: synthesis of carboxymethyl cellulose. *International Journal of Metallurgical and Materials Engineering*.

[26] Sunardi M. F. N., Ahmad B. J. (2017). Preparation of carboxymethyl cellulose produced from purun tikus (Eleocharis dulcis). *AIP Conference Proceedings*.

[27] López G. I. B., Alcudia R. E. R., Veleva L. (2016). Extraction and characterization of cellulose from agroindustrial waste of pineapple (*Ananas comosus* L. Merrill) crowns. *Chemical Science Review and Letters*.

[28] Xu W., Reddy N., Yang Y. (2009). Extraction, characterization and potential applications of cellulose in corn kernels and distillers’ dried grains with solubles (DDGS). *Carbohydrate Polymers*.

[29] Pushpamalar V., Langford S. J., Ahmad M., Lim Y. Y. (2006). Optimization of reaction conditions for preparing carboxymethyl cellulose from sago waste. *Carbohydrate Polymers*.

[30] Phan T. T. M., Cuong H. V., Ho V. C., Pham N. L. (2020). A study on synthesis and properties of SAPs based on carboxymethyl cellulose. *Vietnam Journal of Science, Technology and Engineering*.

[31] Yeasmin S., Akter N., Ahmed N. (2018). A novel optimization method for preparing carboxymethyl cellulose with higher yield from wheat straw. *Journal of Chemical, Biological and Physical Sciences*.

